# Protocol for a Systematic Review of Psychosocial Factors That Predict Mental Health Among Males Living with Type 2 Diabetes

**DOI:** 10.3390/ijerph23070847

**Published:** 2026-06-27

**Authors:** Mokoena Maepa, Mqemane Tshababa, Nkarenbi Juliette Bih, Sello Mantse

**Affiliations:** 1 Department of Clinical Psychology, School of Medicine, Sefako Makgatho Health Sciences University, Pretoria 0204, South Africa; mqemane.tshababa@smu.ac.za (M.T.); sellomantse18@gmail.com (S.M.); 2 Department of Psychology, Faculty of Health Sciences, North-West University (NWU), Mafikeng 2745, South Africa; 28360087@nwu.ac.za

**Keywords:** psychosocial, mental health, men, diabetes, PICO

## Abstract

**Highlights:**

**Public health relevance—How does this work relate to a public health issue?**

- Diabetes and mental health are interconnected and are a public health issue.

**Public health significance—Why is this work of significance to public health?**

- Gender-specific evidence fills the research gap.

**Public health implications—What are the key implications or messages for practitioners, policy makers and/or researchers in public health?**

- Integration improves holistic patient outcomes and Policy supports psychosocial screening integration.

**Abstract:**

Psychosocial factors such as diabetes distress, depression, anxiety, social support, stigma, coping styles, and quality of life play a critical role in shaping mental health outcomes among males living with type 2 diabetes. Despite their importance, systematic evidence on how these factors influence mental health remains limited. Objective: This protocol aims to synthesize existing evidence on psychosocial factors associated with mental health outcomes among males living with type 2 diabetes. Eligibility Criteria: Studies will be included if they focus on males aged 18 years and above diagnosed with type 2 diabetes and examine psychosocial factors in relation to mental health outcomes. Qualitative, quantitative, and mixed-methods designs will be considered. Exclusion criteria include studies focused on females, males under 18, or those with type 1 diabetes; studies exclusively evaluating interventions (e.g., CBT trials, self-management programs); non-English publications; and studies published before 2015. Confounders such as co-morbidities and lifestyle factors will be included if reported alongside psychosocial exposures, but studies focusing solely on these without mental health outcomes will be excluded. Information Sources: The search strategy will be guided by the PICO framework, and such searches will be conducted in Scopus, MEDLINE (PubMed), CINAHL, and Web of Science using Medical Subject Headings (MeSH) for articles published in English between January 2016 and December 2026. Risk of Bias: Two independent reviewers will screen studies, with disagreements resolved by a third reviewer. Risk of bias will be assessed using the JBI Critical Appraisal Checklist. Data Synthesis: Eligible studies will undergo narrative thematic synthesis. Confidence in findings will be evaluated using the JBI ConQual approach. Ethics and Dissemination: Ethical approval is not required. This protocol is registered with PROSPERO (CRD420261299482). Results will be disseminated through peer-reviewed journals and conferences. Conclusion: This protocol outlines a transparent plan to review psychosocial factors influencing mental health in men with type 2 diabetes, guiding gender-sensitive strategies that integrate mental health into diabetes care.

## 1. Introduction

Men living with type 2 diabetes face a complex interplay of psychological and social challenges that predispose them to mental health challenges. One of the most prominent psychological factors is diabetes distress, which refers to the emotional burden of daily self-management. This includes frustration, worry, and burnout, often exacerbated by food restrictions, lack of empathy from family members, and healthcare providers labeling patients as “non-compliant” [1,2,3]. Although it can resemble depression or anxiety, diabetes distress is not a psychiatric illness but rather a stress response to living with a chronic condition.

There is a wide range of psychosocial factors that predispose men living with type 2 diabetes to mental health challenges. Males with type 2 diabetes experience psychological factors like diabetes distress, anxiety and depression [1]. They also go through social factors such as lack of social support, particularly from close family members, the fear of hypoglycemia, a pessimistic life orientation and poor physical and mental quality of life, thus increasing the rates of psychological distress among males living with type 2 diabetes.

As alluded to, one of the psychosocial factors experienced by males living with type 2 diabetes is diabetes distress. According to Riise et al. [2], individuals living with type 2 diabetes (T2D) may experience diabetes distress, commonly referred to as the emotional burden that arises from daily self-management of diabetes. Diabetes distress is described as feelings of frustration and worry that often accompany living with and managing diabetes mellitus [3]. Although a person experiencing diabetes distress may demonstrate depressive and anxious symptoms, diabetes distress is not considered a psychiatric illness but a stress response to living with the condition. Alwani [4] opined that patients living with type 2 diabetes may feel overwhelmed, frustrated, and angry or burnt out due to the burden of living with a chronic disease and the demands of self-management. Food restriction, lack of empathy from family members and the approach that healthcare providers use of labelling patients as non-compliant and scaring them about the grim prospects further worsen distress.

Another important psychological factor in predicting mental health among men living with type 2 diabetes is depression [1]. A cross-sectional study carried out in Denmark by Dalsgaard et al. [5] assessed both diabetes distress and depression. In this Danish study, diabetes was assessed using the Problem Area in Diabetes scale, and depression was assessed via hospital diagnosis and prescribed medication as exposures. Among 18,222 individuals with type 2 diabetes who responded to the survey (46% response rate), 11% were found to have depression, 14% experienced diabetes-related distress, and 4% had both conditions [5]. Interestingly, findings from Dalsgaard et al. [5] revealed that approximately half of those experiencing diabetes distress maintained stable blood glucose levels. The study by Dalsgarard and colleagues used Logistic Regression, adjusting for potential confounders and comparing exposed and non-exposed groups on lifestyle habits, metabolic factors and medication usage related to cardio-metabolic risks. The study by Dalsgarard and colleagues found that those with depression and diabetes had a higher risk of sedentary behavior, clinical insomnia and low self-rated health than those with either psychological condition in isolation. In support, Luo et al. [6] highlight that type 2 diabetes mellitus (T2DM) and depression share a bidirectional relationship that is usually driven by gut–brain axis dysfunction, immune inflation, neuroendocrine dysregulation and metabolic abnormalities. The study by Luo and colleagues concluded that individuals with depression had higher chances of developing T2DM, while those who already have T2DM have a significantly higher prevalence of depression.

In Africa, studies that focused on psychosocial factors that predict the mental health of men living with type 2 diabetes have been carried out, but with a different focus. For example, a study by Sifunda et al. [7] investigated the prevalence of diabetes and its psychosocial correlates in both the general South African population and the Black South African subpopulation using data from the SANHANES-1. Although the Sifunda and colleagues study focused on both males and females, their study has implications for our understanding of psychosocial predictors for mental health and how these affect males living with type 2 diabetes. Another South African study that studied how psychosocial factors affect the mental health of type 2 diabetic patients includes an observational cross-sectional study carried out by Fredericks and Naidoo [8]. This study assessed the quality of care of all patients living with T2DM accessing care in an urban district hospital in Durban, KwaZulu-Natal, South Africa. The study concluded that the quality of care was suboptimal due to poor efficacy indicators, poor knowledge and lack of adequate lifestyle measures, despite the frequency of medical practitioner reviews. A meta-analysis carried out by Nguyen et al. [9] examined the association of depression with glycemic control in adults with diabetes mellitus in low–middle income countries (LMICs). The study found that the burden of uncontrolled diabetes and diabetes complications correlated with higher levels of depression.

Studies have repeatedly demonstrated the connection between social connection and better treatment outcomes, disease management and the overall health of males living with either type 1 or type 2 diabetes [10,11]. The social connection is usually in the form of family support and family involvement. To buttress the role of social connection, a study by Holt-Lunstad [12] concluded that people with smaller social networks who have been diagnosed with type 2 diabetes are likely to have poor mental health outcomes due to diabetic complications. Holt-Lunstad’s meta-analysis of 28 studies found that social support was significantly associated with better self-care, particularly glucose monitoring, and was stronger among those with type 2 than type 1 diabetes. In the same study, Holt-Lunstad suggests that poor social connections are linked to poor health outcomes among those who already have either type 1 or type 2 diabetes.

Other studies that looked at psychosocial factors as predictors of mental health among males living with type 2 diabetes focused on social issues like quality of life. Regarding quality of life, Kien et al. [13] coined the health-related quality of life (HRQoL) concept. This concept describes the individual’s views of their emotional, physical and social wellbeing, where poor quality of life predisposes individuals living with type 2 diabetes to mental health complications and where good quality of life improves individuals’ mental well-being. All these noted psychosocial factors related to diabetes necessitate the need for mental health interventions.

Some interventions are critical in the management of mental health issues in males living with type 2 diabetes. Voeltz et al. [14] identify training on self-management as a critical intervention in controlling the psychosocial factors and their contribution to poor mental health. Self-care is important because it recognizes the role of the patient as an active participant in his or her own care plans and treatment. According to Van Smoorenburg et al. [15], self-management comprises three distinct tasks, and these are medical management, behavior management, changing and adopting new behaviors and emotional management.

Studies such as Clarke et al. [16] and Cummings et al. [17], as cited in Voeltz et al. [14], identify Cognitive Behavioral Therapy (CBT) as a key intervention strategy to manage mental health issues in males living with type 2 diabetes. The authors argue that CBT helps in improving the treatment of mood disorders in individuals with type 2 diabetes. In addition, strategies such as comprehensive screening can be adopted where professionals screen for issues like anxiety and depression. The adoption of comprehensive screening allows relevant treatment and management options for men living with type 2 diabetes to be put in place. Other key interventions that might help to manage the mental health challenges among men living with type 2 diabetes may include engaging in counselling, motivational interviews and the provision of health education. Providing health education to males living with type 2 may change their perspectives on behavior change to adopt more positive ways of handling their diabetes, as well as their mental health.

The rationale for this study lies in the need to better understand how psychosocial factors uniquely affect men living with type 2 diabetes. While global research has established links between diabetes distress, depression, and social support, there is limited exploration of how these factors intersect specifically in male populations, particularly within African contexts. Addressing this gap is essential for developing gender-sensitive and culturally relevant interventions.

The novelty of the study is threefold. First, it emphasizes a gender-specific focus, recognizing that men may experience distinct psychosocial stressors shaped by cultural expectations and masculinity norms. Second, it integrates multiple psychosocial domains—distress, depression, social support, and quality of life—rather than examining them in isolation, offering a holistic perspective. Third, it situates findings within African contexts, contributing region-specific insights that are often missing in the global diabetes literature. Finally, by acknowledging both psychosocial and biological mechanisms such as gut–brain axis dysfunction, the study bridges biomedical and psychosocial perspectives, advancing a more comprehensive understanding of mental health in men with type 2 diabetes.

The primary goal of this systematic review protocol is to outline the methodological approaches and literature search strategies that will be employed to conduct a systematic review on psychosocial factors influencing mental health among males with type 2 diabetes. The protocol will gather information on psychosocial predictors of mental health in this demographic. It will also cover the central research questions addressed by the review, a detailed explanation of the systematic literature search methods, criteria for study inclusion or exclusion, a description of the coding procedures, assessments of study quality, and the statistical techniques used for the quantitative analysis of data from the studies that meet the eligibility criteria.

## 2. Materials and Methods

### 2.1. Reporting Guidelines

The systematic review shall be conducted in adherence to the Preferred Reporting Items for Systematic Reviews and Meta-Analysis (PRISMA) and the Consolidated Standards of Reporting Trials (CONSORT) guidelines. The PRISMA 2020 [18] checklist will be used to guide the review structure, thus ensuring complete, transparent and accurate reporting. This protocol is registered and published with the International Prospective Register of Systematic Reviews (PROSPERO) database under registration number CRD420261299482.

### 2.2. Research Questions

What psychosocial factors are associated with mental health outcomes among males living with type 2 diabetes?

Sub research questions:

What are the specific psychosocial factors that have been studied in relation to males living with type 2 diabetes?

Are certain psychosocial factors more strongly associated with mental health outcomes than others are?

What mental health outcomes are usually reported for males living with type 2 diabetes?

Do psychosocial interventions improve the mental health of males with type 2 diabetes?

### 2.3. Eligibility Criteria

The inclusion and exclusion criteria for the proposed study are based on the Population, Intervention, Comparisons, Outcome, and Study Type (PICOS) framework as indicated in Table 1 below. As alluded to, regarding the population, all studies on males from the age of 18 years upwards with type 2 diabetes will be considered for the systematic review. Further, only peer-reviewed studies in full text, or doctoral dissertations are eligible for this review, and only studies written in English shall be included. Regarding the intervention, studies that have examined any psychological or educational support meant to promote mental health in males living with type 2 diabetes will be included, while those that focused on both males and females will be excluded from this research. Confounders such as co-morbidities and lifestyle factors will be included if reported alongside psychosocial exposures, but studies focusing solely on these without mental health outcomes will be excluded. In terms of comparisons, no comparison group will be required for this systematic review. In terms of the outcomes, studies that focus on psychosocial factors that either improve or worsen the mental health of males living with type 2 diabetes will be included in this systematic review. Studies that focus on anxiety, diabetes distress and depression will be looked at. In terms of the study design, the proposed study will use a quantitative approach for studies with comparable designs.

Importantly, only studies written in English will be included.

#### 2.3.1. Exclusion Criteria

- Studies focused on females, males under the age of 18, or males diagnosed with type 1 diabetes.
- Studies that exclusively evaluate interventions (e.g., CBT trials, self-management programs).
- Non-English publications.
- Studies published before 2015.

#### 2.3.2. Confounders

The inclusion or exclusion of confounders are to ensure clarity in synthesis:

Co-morbidities such as hypertension, cardiovascular disease, and obesity will be included if clearly reported, but studies exclusively focused on these conditions without reference to psychosocial factors will be excluded.

Lifestyle factors such as smoking, alcohol use, physical activity, and diet will be considered if they are reported as part of psychosocial predictors. Studies focusing solely on lifestyle interventions without mental health outcomes will be excluded.

### 2.4. Information Sources

All authors shall conduct a systematic search of the literature, and the search shall be carried out electronically on databases such as Web of Science, Scopus, PubMed, Embase and PsycINFO. Additionally, searches will also be extended to databases of academic and research institutions such as the National Institutes of Health (NIH) and through browsing articles on Google Scholar and Research Gate for studies published within the last decade (January 2015–December 2025).

### 2.5. Search Strategy

The PICO framework will be used to guide the literature search. We aim to create an extensive database that includes all published research examining psychosocial factors as predictors of mental health in males with type 2 diabetes. To compile a comprehensive list of potential search terms, we will note indexing terms like subject headings and subheadings, as well as publication types, alongside keywords and phrases used to denote concept clusters, which may appear in titles or abstracts in both complete and abbreviated forms. The search strategy will include the identification of main terms as per the PICO framework. Words and phrases such “psychosocial factors’’ OR “Psychological factors” OR “Social factors” AND Predictors AND “Mental Health” OR Mental OR Health AND “Type 2 Diabetes” OR Diabetic OR Diabetes OR Insulin AND men OR Males will be used in their singular or plural in the searches, either in titles, abstracts, keywords or text fields of the journal articles.

### 2.6. Data Management

We plan to carry out the search strategies and transfer all identified references to EndNote using Rayyan (Rayyan System Inc., Cambridge, MA, USA), an online tool that aids in reference selection for systematic reviews by screening the identified literature. The search outcomes from various electronic databases will be merged into one EndNote library (Clarivate Analytics, Philadelphia, PA, USA), and we will eliminate duplicate entries of the same reports.

## 3. Selection Process

The first and second authors shall be responsible for the procedure selection and shall be guided by the inclusion and exclusion criteria previously outlined. They will individually assess titles and abstracts based on eligibility criteria to pinpoint studies that could be included. The articles identified using titles, abstracts, and keywords will be reviewed by two authors and shall be characterized as included, excluded and maybe. The full articles will be similarly retrieved, and the third author will carry out further reviews to check for consensus among reviewers and to provide resolutions in areas of divergence.

**Figure 1:**
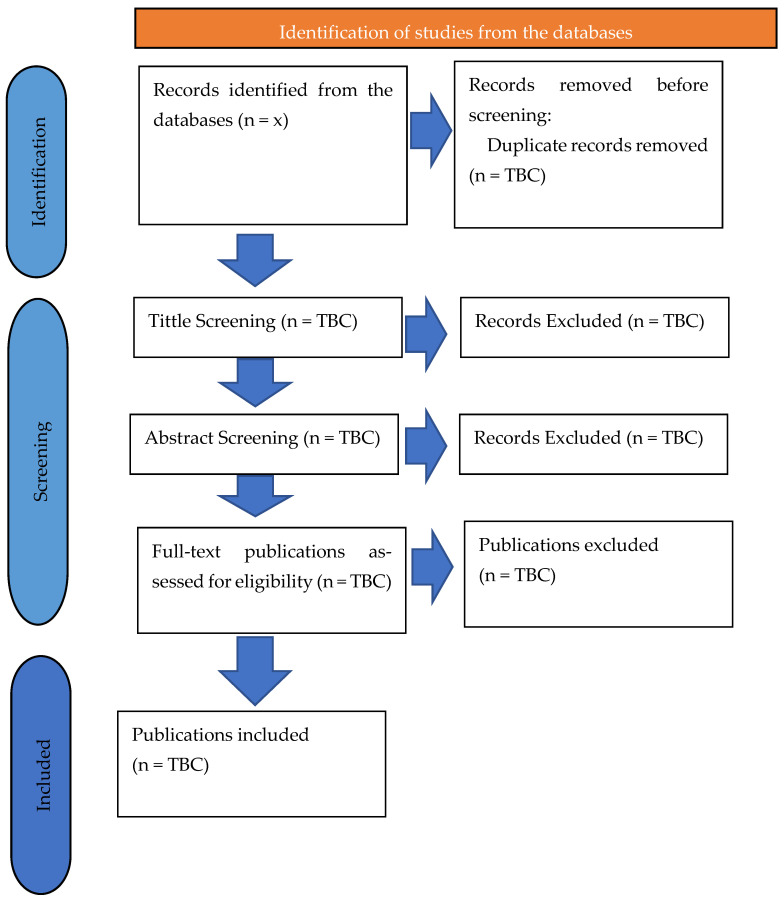
PRISMA 2020 flow diagram.

The selection process will be guided by the PRISMA 2020 flow diagram for updated systematic reviews, which will include searches across all databases as depicted in Figure 1 below.

### 3.1. Data Collection Process

To reduce bias and ensure objectivity in the extraction of data, the first author and the second author will independently screen, extract and manage the data for each of the included studies. Authors will examine all data, and any disagreements will be resolved through discussion. The third author will act as an arbiter in case disagreements cannot be resolved by the first and second authors. More so, authors three and four will evaluate and validate the extracted data.

### 3.2. Data Items

Depending on availability, the following data shall be extracted from the identified studies: the information concerning the article, the information about the participants, the information about the features of the study, the information about data collection, and the conclusions of the study.

Publication details: title, journal, author, year, city and country in which the study was conducted.

- Design: type of study (observational studies, cross-sectional, cohort, case–control, single arm studies, systematic review/meta-analyses), aims of the study, method of data collection, response rate, recruitment and sampling methods, and eligibility (inclusion and exclusion criteria).
- Study participant details: number of persons interviewed or surveyed and population characteristics, including setting, age, gender, ethnicity, and demographic information.
- Data for outcome measures: all reported estimates, or sufficient information on psychosocial factors as predictors of mental health among males living with type 2 diabetes.
- Limitations: selection bias, response bias, information bias, limitations of assessment tool(s) used, and limitations reported by study authors.
- Table 2 below displays the data extraction.

### 3.3. Outcomes and Prioritization

This systematic review seeks to determine the extent to which psychosocial factors predict the mental health condition of male adults living with type 2 diabetes. The review seeks to identify those psychosocial factors that negatively affect the mental health of males living with type 2 diabetes. Factors such as diabetes distress, quality of living, depression, self-care and social connection will be examined to establish the extent to which they affect the mental health of males living with type 2 diabetes.

### 3.4. Methodological Quality Assessment in Individual Studies (Risk of Bias)

The risk of bias in the included studies will be assessed using the PRISMA 2020 recommendations to ensure quality of assessment in identified papers [18]. Other complementary tools, such as the Joanna Briggs Institute (JBI) Critical Appraisal Tool (Joanna Briggs Institute, Faculty of Health and Medical Sciences, The University of Adelaide, Adelaide, South Australia, Australia), shall be used to reduce the risk of bias. The JBI Critical Appraisal Checklist for Analytical Cross-Sectional Studies tool will be used. The instrumental framework assesses factors like representativeness, recruitment, sample size, reporting, data coverage, condition reliability, statistical analysis, and confounding elements using a straightforward response system of “yes”, “no”, “unclear”, or “not applicable”. The checklist includes items with responses such as “yes”, “unclear”, and “not applicable”. To address publication bias and selective reporting, study findings will be critically evaluated. Additionally, plots will be drawn of outcome variables against sample size following Egger et al. [17], and guidance will be sought from GRADE guidelines No 5 [19]. The decisions regarding the review will be made based on the inter-rater agreement (Cohen’s kappa), which shall be completed by two reviewers. All disagreements and conflict issues will be resolved through discussions. If there is no consensus after discussions, a third reviewer will be sought for consensus.

### 3.5. Data Synthesis

The data will be synthesized following a systematic narrative synthesis. The synthesized findings from studies will be presented in the word and text format, accompanied by summaries that explain the findings of the synthesis. The narrative synthesis analysis is important when integrating various types of evidence and in understanding the associations between studies. The narrative synthesis will summarize psychosocial factors as predictors of mental health in males with type 2 diabetes, categorized by age and setting. These grouping variables will aid in identifying similar patient populations, facilitating quantitative meta-analyses for studies with comparable designs. The extracted data will be displayed with corresponding standard error and 95% confidence intervals using the exact binomial method, as detailed by Clopper and Pearson [20]. This method yields an exact confidence interval based directly on the binomial distribution, rather than an approximation of it. The data synthesis of this systematic review will follow three steps, namely the coding of text, the development of descriptive themes and generating analytical themes. The systematic narrative synthesis shall be based on the method as recommended by Popay et al. [21] that emphasizes a transparent and systematic way of analysis.

## 4. Discussion

Understanding the role of psychosocial factors on the mental health of men living with type 2 diabetes is of paramount importance. The methodology outlines a systematic review protocol aimed at identifying psychosocial factors associated with mental health outcomes among males living with type 2 diabetes. This systematic review followed the PRISMA guidelines, and the protocol was registered in PROSPERO (CRD420261299482.) to ensure that there is no duplication of studies. All searches for keywords and issues will be conducted systematically until data saturation is reached. The review seeks to answer questions related to psychosocial predictors of mental health, common mental health outcomes experienced by males with type 2 diabetes, and the effectiveness of psychosocial interventions within this population.

The study will include peer-reviewed quantitative studies and doctoral dissertations published in English between 2015 and 2025. Eligible participants will include males aged 18 years and older diagnosed with type 2 diabetes. Studies focusing on females, minors, type 1 diabetes, or interventions without mental health outcomes will be excluded. Psychosocial variables such as social support, diabetes distress, depression, anxiety, quality of life, self-care, and social connection will be explored in relation to mental health outcomes.

A comprehensive electronic literature search will be conducted using databases including PubMed, Scopus, Web of Science, Embase, PsycINFO, Google Scholar, and ResearchGate. Search terms will be guided by the PICO framework and will include combinations of psychosocial factors, mental health, type 2 diabetes, and males. Retrieved studies will be managed using EndNote and screened collaboratively through Rayyan by independent reviewers using predefined inclusion and exclusion criteria.

Data extraction will focus on study characteristics, participant demographics, psychosocial variables, interventions, mental health outcomes, and study findings. Methodological quality and risk of bias will be assessed using PRISMA recommendations and Joanna Briggs Institute critical appraisal tools. Reviewer agreement will be evaluated using Cohen’s kappa statistic.

Due to anticipated heterogeneity across studies, findings will be synthesized using a systematic narrative synthesis approach rather than meta-analysis. The synthesis will involve coding extracted data, developing descriptive themes, and generating analytical themes to identify psychosocial predictors of mental health among males living with type 2 diabetes.

## 5. Conclusions

This systematic review protocol outlines the proposed methodological approach for synthesizing evidence on psychosocial factors associated with mental health outcomes among males living with type 2 diabetes. The protocol was developed to ensure methodological transparency, rigour, and reproducibility throughout the review process. By applying structured eligibility criteria, comprehensive database searching, independent screening procedures, and systematic narrative synthesis, the proposed review aims to provide a comprehensive understanding of psychosocial determinants influencing mental health within this population.

The findings of the planned systematic review are expected to contribute toward identifying key psychosocial factors associated with psychological well-being among males living with type 2 diabetes, while also highlighting existing knowledge gaps within the literature. Furthermore, the review will inform future research, clinical practice, policy development, and the design of contextually relevant psychosocial interventions aimed at improving mental health outcomes among males living with type 2 diabetes.

## Figures and Tables

**Table 1:** Studies to be included in the review in line with the PICO framework.

PICO	Criteria
Population	All males aged 18 years and above. Patients with type 2 diabetes. No restriction to the country of origin, cultural background or the socio-economic status.
Interventions	Any psychological or educational support meant to promote mental health.
Comparators	The study does not focus on comparative groups.
Outcomes	Mental health outcomes (depression, anxiety, distress, wellbeing, quality of life).

**Table 2:** Data items to be extracted from selected articles.

Category	Data Items to be Extracted
Study Information	Authors Year of publication City Country Study type
Country Economic Status	Developing and developed countries
Study Question(s)	Aim of the study Research question (s)
Participant Characteristics	Age Ethnicity
Study Features	Study setting Study design Inclusion/Exclusion design Sample size Assessment points
Intervention Features	Type of intervention protocol Description
Measures	Diabetes distress, social support, anxiety, depression
Outcomes	Mental health outcomes (depression, anxiety, distress, wellbeing, quality of life)
Main Results of the Study	Association between psychosocial factors and mental health Prevalence of poor mental health among men living with type 2 diabetes

## Data Availability

The data sets generated for this study will be available at figshare.
